# Individual Differences in Serial Dependence of Facial Identity are Associated with Face Recognition Abilities

**DOI:** 10.1038/s41598-019-53282-3

**Published:** 2019-12-02

**Authors:** Kaitlyn Turbett, Romina Palermo, Jason Bell, Jessamy Burton, Linda Jeffery

**Affiliations:** School of Psychological Science, University of Western Australia, Crawley, WA Australia

**Keywords:** Psychology, Visual system

## Abstract

Serial dependence is a perceptual bias where current perception is biased towards prior visual input. This bias occurs when perceiving visual attributes, such as facial identity, and has been argued to play an important functional role in vision, stabilising the perception of objects through integration. In face identity recognition, this bias could assist in building stable representations of facial identity. If so, then individual variation in serial dependence could contribute to face recognition ability. To investigate this possibility, we measured both the strength of serial dependence and the range over which individuals showed this bias (the tuning) in 219 adults, using a new measure of serial dependence of facial identity. We found that better face recognition was associated with stronger serial dependence and narrower tuning, that is, showing serial dependence primarily when sequential faces were highly similar. Serial dependence tuning was further found to be a significant predictor of face recognition abilities independently of both object recognition and face identity aftereffects. These findings suggest that the extent to which serial dependence is used selectively for similar faces is important to face recognition. Our results are consistent with the view that serial dependence plays a functional role in face recognition.

## Introduction

The ability to learn and recognise faces is a crucial component of social interaction and communication. Although adults are generally considered to be experts at determining an individual’s identity, individual differences in this ability are well documented^1^. The variation in face recognition abilities has been argued to reflect individual differences in face-processing mechanisms such as holistic coding, where features of a face are simultaneously integrated to produce a single perceptual representation^2^, with only small contributions from general cognitive abilities^3–10^, but see^11^.

Intriguingly, variation in face recognition ability has also been associated with the strength of perceptual biases in face perception. Face identity aftereffects are a type of perceptual bias in which adaptation (exposure) to a face’s identity distorts subsequently viewed faces, such that perception of identity is biased away from the characteristics of the adaptor face^12^. There are individual differences in the strength of face identity aftereffects, and these are found to be associated with individual differences in face recognition ability, such that stronger face identity aftereffects are associated with better face recognition abilities^3–5^. This relationship may appear counterintuitive, but the perceptual bias induced by adaptation is thought to optimise sensitivity to novelty by biasing perception away from the adapted state^13^. Through adaptation, any subsequent changes in the environment are then viewed as more distinct. For faces in particular, aftereffects are also argued to reflect adaptive norm-based coding, whereby subtle variations in the attributes of a face are coded relative to norms that are constantly revised and updated to reflect the average attributes of encountered faces^14^. Therefore, individual variation in the strength of aftereffects is thought to reflect the ease with which these norms are calibrated by experience^15^. Consistent with this view that face aftereffects reflect an important perceptual mechanism of face perception, face identity aftereffects are not related to object recognition abilities and the relationship between face identity aftereffects and face recognition remains significant when controlling for non-face object recognition^3–5^. These results, along with other evidence^16,17^, suggest that face identity aftereffects are operating at the level of facial identity.

Here we ask whether another perceptual bias, serial dependence, may also contribute to variation in face recognition ability. Serial dependence occurs where current visual perception is biased towards what has previously been seen. In contrast to adaptation, this means that an object is perceived to be more similar to a prior object than it really is^18^. Serial dependence is thought to serve a complementary function to adaptation, promoting perceptual stability in the visual system^19^. Individuals need to perceive objects as stable and unchanging, despite fluctuations in visual input (e.g. from eye movements, lighting changes, occlusion). Given that brief changes in an object over a short period of time are more likely to be due to temporary neural fluctuations rather than changes in the object itself, serial dependence is argued to improve signal-to-noise ratio and perceptual stability for objects over time by integrating information across several “noisy” percepts^18–23^. For this reason, serial dependence appears to occur most readily when exposed to a series of visual inputs that are brief in duration and somewhat ambiguous, as opposed to adaptation which generally occurs after relatively longer exposures^18^.

Serial dependence has been found for the perception of a variety of visual characteristics and objects, leading to suggestions that this bias reflects a general visual processing principle^20^. In low-level vision, serial dependence has been found for stimuli orientation^18,22,24,25^ and numerosity^26^. Recent studies show that serial dependence extends to behaviourally relevant and complex objects, like bodies^27,28^ and faces. Within faces, serial dependence has been found for judgments of attractiveness^29–31^, gender^32^ and facial identity^33,34^.

Serial dependence of facial identity occurs where the perceived identity of a face is biased towards the identity of a previously presented face, such that the second face looks more like the first than it really is^33^. Serial dependence for facial identity has been found to operate across changes in viewpoint^33^, showing that this bias integrates an individual’s identity despite changes in lower-level image features. Importantly, individual differences in the strength of serial dependence for face identity have been found^35^. Given this individual variability and the evidence that serial dependence can operate on high-level facial identity information, it is possible that variation in this bias contributes to face recognition ability. Here we investigate this possibility by examining whether there is a relationship between serial dependence of facial identity and face recognition abilities, consistent with serial dependence playing a functional role in face perception.

If stability is important for face identity recognition, then we might predict that stronger serial dependence would be linked with better face identity recognition skills. Two lines of research support this position. First, there is evidence that serial dependence operates on stable attributes rather than more changeable ones. Face identity is a stable attribute of a face that does not change from moment to moment within an individual (along with gender and attractiveness), in contrast to other, more changeable, aspects of a face that can vary often and quickly (such as expression and eye gaze). When individuals are required to judge the gender and expression of a face simultaneously, gender was biased towards the previous face while expression was biased away from the previous face, suggesting that it may be better to integrate more stable attributes through serial dependence and maximise sensitivity to change for more changeable attributes through aftereffects^32^. As serial dependence appears to selectively operate on stable attributes, it may be that integration is functionally beneficial to recognising stable attributes over more changeable ones.

Second, serial dependence could also contribute to building a stable representation of an individual’s identity. Previous research has found that individuals are able to create an average identity from sequentially presented images of an identity, and this average is argued to contribute to building a stable representation of an individual^36^. Serial dependence could facilitate the creation of this average face and, therefore, a more stable and robust representation of a given individual. Individuals who have stronger serial dependence could therefore form more robust representations, leading to better face recognition abilities.

However, it is also possible that stronger serial dependence could make it more difficult for an individual to recognise an identity. As noted earlier, better face recognition abilities are associated with stronger face identity aftereffects. That is, a stronger bias to perceive two consecutive face stimuli as different is associated with better face recognition skills. This bias can be thought of as facilitating discrimination between individuals, which is also crucial for face recognition. In contrast when individual identities are assimilated via the process of serial dependence, the fine details that make a face more distinct may be lost through this integration process, such that serial dependence may make individual discrimination more difficult. This integration may be particularly problematic when integrating stimuli that are more distinct and dissimilar and has been suggested to result in large errors in estimation^19^, and could potentially result in the fine details needed to tell individuals apart being integrated and smoothed over. Therefore, if individuals integrate identities that are more distinct from one another, this could be problematic for recognition.

Therefore, to understand the functional role of serial dependence it is important to not only consider the strength of serial dependence, but also how the strength of this bias varies depending on the difference between consecutive stimuli. Cicchini *et al*.^19^ recently showed that serial dependence for orientation is strongest when two sequential stimuli are more similar to one another, as well as when the stimuli are more ambiguous (and individuals are less certain about the properties of the stimuli that they are asked to judge). This evidence suggests that serial dependence (for both orientation and body perception) may be tuned to the degree of similarity between successive stimuli, operating most strongly when stimuli are very similar^19,27^. This leads to the possibility that there may be individual differences in the tuning of serial dependence, so that having narrower tuning (i.e., predominantly using serial dependence for more similar stimuli) may benefit face recognition, while having broader tuning (i.e., using serial dependence for all stimuli regardless of their similarity) may make it more difficult to discriminate and recognise faces.

In the current study, we used an individual differences approach to examine whether variation in serial dependence contributes to face recognition abilities, in either a beneficial or a detrimental way. We measured individual differences in serial dependence for facial identity using a novel task, based on Fischer and Whitney’s^18^ design, in which participants were trained to recognise four identities, two males (‘Tim’ and ‘Jon’) and two females (‘Mel’ and ‘Sue’). The strength of this bias was measured for each participant. In addition, to assess the tuning of serial dependence, we manipulated the similarity of the two faces in each pair, with the prediction that serial dependence would be strongest when stimuli are more similar to one another^19,27^ but that the degree to which this occurred might vary amongst participants. Face identity recognition abilities were measured using the Cambridge Face Memory Test (CFMT)^37^, a valid and reliable measure of individual differences in face memory recognition ability^7,38^. We also included the Cambridge Car Memory Test (CCMT)^39^ to control for general object recognition and thereby determine whether any observed effects were unique to face recognition, or rather reflect more general visual recognition^3–5^. Finally, we included a face identity aftereffect task^5^ to examine whether serial dependence contributed to face recognition abilities independently of face identity aftereffects.

## Results

### Calculation of serial dependence values

On each trial, two faces from a morph continuum (i.e., ‘Tim’ and ‘Jon” or ‘Mel’ and ‘Sue’) were presented sequentially and participants were required to identify the second face that appeared. On each trial, we were interested in the influence of the first face on the perceived identity of the second. If serial dependence occurred, the second face would be more likely to be perceived as a given identity when it was preceded by a face more similar to that identity (e.g. more likely to perceive the second face as Jon when it was preceded by a more Jon-like face).

Within each trial, the two faces presented could differ from each other in one of four morph difference intensities: −24%, −12%, +12%, or +24%. For each of these four conditions, the proportion of Jon responses (or Sue for female trials) was calculated. Differences of −24% and −12% between Face 1 and Face 2 indicate Face 1 was less like Jon/Sue while differences of +12% and +24% indicate that Face 1 was more like Jon/Sue. Serial dependence was expected to bias perception towards the previously seen identity, so we expected a greater proportion of Jon/Sue responses to Face 2 when Face 1 resembled Jon/Sue (i.e., differed by +12% and +24%) and a smaller proportion of Jon/Sue responses to Face 2 when Face 1 resembled Tim/Mel (i.e., differed by −24% and −12%).

#### Estimating serial dependence strength

**Figure 1:**
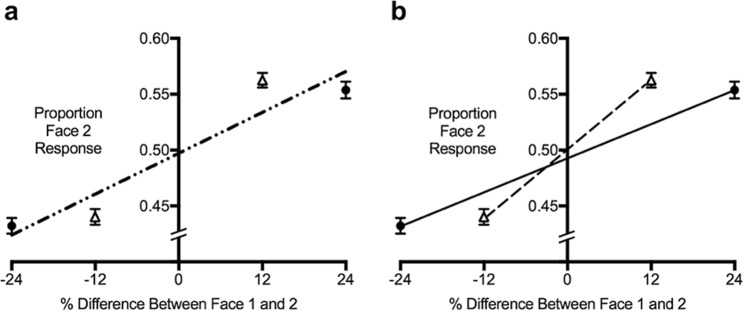
Mean responses as a function of the percentage difference between Face 1 and Face 2. Regression slopes fit to the group means for illustrative purposes. A larger, positive gradient indicates stronger serial dependence. Figure (**a**) shows the linear regression fit to all data points, slope = 0.0030, *R*^2^ = 0.89. Figure (**b**) shows the linear regressions fit separately for when Face 1 and 2 were more similar (±12%. Inner triangle data points and dashed line, slope = 0.005, *R*^2^ = 1.0) and for when they were more dissimilar (±24%. Outer circle data points and solid line, slope = 0.003, *R*^2^ = 1.0). Error bars represent standard error. Serial dependence was significantly larger when Face 1 and 2 were more similar (±12% trials; *M* = 0.005, *SD* = 0.004) compared to when they were dissimilar (±24% trials; *M = *0.002, *SD = *0.003), *t*(218) = 9.42, *p* < 0.001, *d* = 0.64. The significant difference between serial dependence for similar and dissimilar faces is further evidence of the non-linearity of serial dependence in our sample and supports treating the strength of this bias for similar and dissimilar faces separately.

Previous research has found a non-linear pattern of serial dependence, in that serial dependence is stronger when visual stimuli are more similar and decreases in strength as stimuli become more distinct^18,19,27^. As can be seen in Fig. 1, this non-linear pattern was also present in our group data. Consequently, fitting a linear regression to all four data points is likely to provide an underestimate of the strength of serial dependence. Therefore, consistent with previous research^27^, we calculated serial dependence strength based on the ±12% trials, as this was when Face 1 and Face 2 were closer together on the morph continuum and therefore more similar to one another. To calculate the strength of serial dependence (SDFI_strength) a linear regression was fit to the proportion of Jon/Sue responses for ±12%. A positive gradient indicated that serial dependence occurred, and individuals were biased towards the previous identity, a value of zero indicated no serial dependence, while a negative value indicated that individuals were biased away from the previous identity, consistent with adaptation.

#### Estimating serial dependence tuning

**Figure 2:**
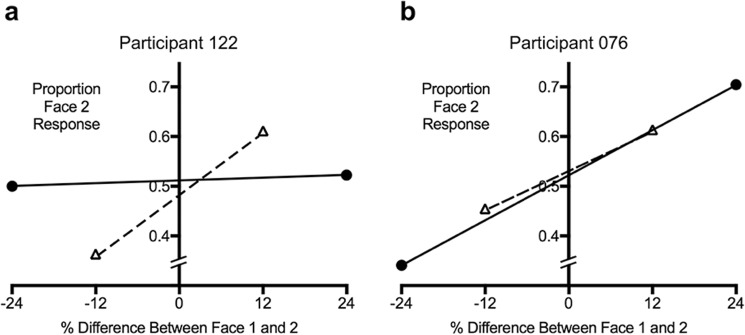
Two examples demonstrating differences in the tuning of serial dependence. The triangles represent the responses for when Face 1 and Face 2 were more similar to one another (varied by ±12%) and the dashed line is the linear regression fit to these values. The circles and solid black line represent the responses for when Face 1 and Face 2 were more dissimilar to one another (varied by ±24%) and the linear regression fit. Figure (**a**) shows a greater difference score between the slope of the outer two points and inner two points, which indicates narrower tuning of serial dependence (difference score = 0.010), suggesting that serial dependence was used to a greater extent when the two faces were more similar. Figure (**b**) shows a smaller difference score, which indicates broader tuning of serial dependence (difference score = −0.001) as the use of serial dependence scaled linearly with face dissimilarity. While both participants show similar strength of serial dependence (dashed lines), the tuning value shows that they vary in the range that they used serial dependence.

While as a group, participants showed a non-linear pattern of serial dependence, individual participants varied in their use of serial dependence (see Fig. 2 for examples). For example, while both participants in Fig. 2 show a similar strength of serial dependence, they differ substantially in the range across which serial dependence occurs. Some individuals showed a pattern of stronger serial dependence when Face 1 and Face 2 were more similar, and weaker serial dependence when the faces were less similar (Fig. 2a). Others showed a pattern of serial dependence that scaled linearly with face dissimilarity, in that use of serial dependence increased as face dissimilarity increased (Fig. 2b). Therefore, we calculated a tuning value to characterise the range that each individual used serial dependence depending on how similar Face 1 and 2 were. This value quantified whether serial dependence was greatest for more similar stimuli, or whether it was used across a broader range of faces and the extent that this occurred. The tuning value (SDFI_tuning) was calculated by subtracting the gradient for the dissimilar faces (calculated by fitting a linear regression to the proportion of Jon/Sue responses on the ±24% trials) from the gradient for the similar faces (±12%). A larger, positive tuning value indicates that there is greater serial dependence for similar faces than dissimilar faces (narrower tuning, see Fig. 2a) and a value closer to 0 or a negative value indicates serial dependence increased as dissimilarity increased (broader tuning, see Fig. 2b).

### Preliminary data analyses

#### Distribution

As suggested by Hoaglin and Iglewicz^40^, we defined univariate outliers as scores 2.2 times the interquartile range but none were identified. All variables were normally distributed (skew less than |2| and kurtosis less than |9|)^41^. No multivariate outliers were identified according to Cook’s distance. Descriptive statistics are reported in Table 1.

**Table 1:** Descriptive statistics for the Serial Dependence of Facial Identity Task, Face Identity Aftereffect task, and Face and Nonface Recognition tasks.

Task	*N*	Min	Max	Mean	*SD*	Skew	Kurtosis
SDFI_strength	219	−0.009	0.018	0.005	0.004	−0.225	−0.062
SDFI_tuning	219	−0.011	0.012	0.003	0.004	−0.314	−0.158
FIAE	209	−0.438	0.750	0.234	0.219	−0.008	−0.097
CFMT_total	219	45.83	100.00	78.13	11.75	−0.321	−0.661
CCMT_total	219	44.44	100.00	69.76	12.91	0.296	−0.472

Note. SDFI = Serial Dependence of Facial Identity Task, FIAE = Face Identity Aftereffect Task, CFMT = Cambridge Face Memory Test, CCMT = Cambridge Car Memory Test.

#### Gender differences

Consistent with previous research^3^, men performed significantly better than women on the car memory test, *t*(216) = 3.69, *p* < 0.001. To control for the influence of gender, we saved the unstandardised residuals from regressing the car test on gender. This residualised score (CCMT_residuals) was then used in all analyses involving the car memory test. For all other measures, no gender differences were found, and analyses were collapsed across gender.

#### Serial dependence of facial identity strength

To determine whether our sample showed significant serial dependence, we compared the mean gradient from the ±12% trials to zero (which would indicate no serial dependence). A one-sample *t*-test indicated that the strength of serial dependence was significantly different from zero, *t*(218) = 16.91, *p* < 0.001, *d* = 1.14, indicating the task produced a significant effect of serial dependence at the group level.

#### Face identity aftereffect

The aftereffect exhibited by participants was significantly different to chance performance that would be expected if there was no effect of adaptation (0.00), *t*(208) = 15.43, *p* < 0.001, *d* = 1.07. Therefore, as a group, participants showed a significant face identity aftereffect.

### Is serial dependence associated with face recognition abilities?

**Figure 3:**
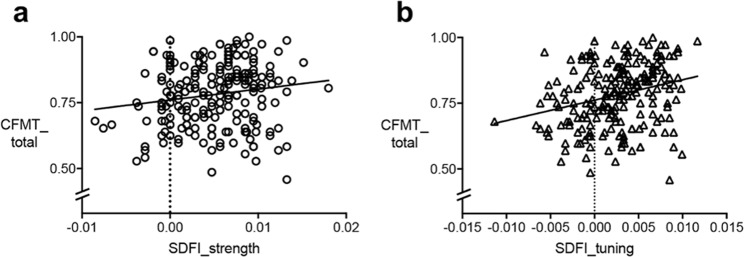
Relationships between serial dependence of facial identity and face recognition abilities. Figure (**a**) shows the relationship between serial dependence of facial identity strength and face recognition abilities (*r* = 0.157 *p* = 0.020), where stronger serial dependence of facial identity was associated with better face recognition abilities. Figure (**b**) shows the relationship between serial dependence tuning and face recognition abilities (*r* = 0.266, *p* < 0.001), where more narrow tuning, or using serial dependence preferentially for when two faces are more similar over when they are dissimilar, was associated with better face recognition abilities.

Our first aim was to examine whether the strength of serial dependence was significantly associated with face recognition abilities as measured by the CFMT. Serial dependence strength was significantly and positively associated with face recognition abilities *r* = 0.157 *p* = 0.020 (see Table 2 and Fig. 3a). No significant relationship was found between serial dependence strength and object recognition abilities. However, the relationship between serial dependence strength and face recognition abilities was not significantly stronger than the relationship between serial dependence strength and object recognition (*Z* = 0.072, *p* = 0.471).

**Table 2:** Pearson correlations between all measures.

	SDFI_strength	SDFI_tuning	FIAE	CFMT_total	CCMT_residuals
SDFI_strength	1				
SDFI_tuning	0.720**	1			
FIAE	0.041	0.100	1		
CFMT_total	0.157*	0.266**	0.173*	1	
CCMT_residuals	0.099	0.106	0.025	0.286**	1

Note. *N = *219 (209 for correlations involving FIAE); *p < 0.05 (two-tailed); **p < 0.001 (two-tailed); SDFI = Serial Dependence of Facial Identity Task, FIAE = Face Identity Aftereffect Task, CFMT = Cambridge Face Memory Test, CCMT = Cambridge Car Memory Test.

Our second aim was to assess whether the tuning of serial dependence, the variation in the strength of this bias depending on how similar Face 1 and 2 were, was significantly associated with face recognition abilities. The estimate of tuning of serial dependence was significantly, positively associated with face recognition abilities (*r* = 0.266, *p* < 0.001; see Fig. 3b). This indicates that more narrow tuning, or using serial dependence preferentially for when two faces are more similar over when they are dissimilar, is associated with better face recognition abilities. While the relationship between serial dependence tuning and object recognition abilities was small and positive (*r* = 0.106, *p* = 0.117), it was not significant. Further, the relationship between serial dependence tuning and face recognition abilities was significantly stronger than the relationship between serial dependence tuning and object recognition (*Z = *2.017, *p = *0.044). Overall, this suggests that the relationship between serial dependence of facial identity tuning and face recognition abilities is specific to face recognition abilities rather than general recognition abilities.

### Does serial dependence of facial identity contribute to face recognition abilities, independently of face identity aftereffects?

A final question was to determine whether serial dependence of facial identity contributed to face recognition abilities, independently of face identity aftereffects. Before examining the contribution of these perceptual biases to face recognition, we first assessed the relationship between face identity aftereffects and both face recognition abilities and serial dependence. As expected^3,4^, we found a significant relationship between face identity aftereffects and face recognition abilities (*r* = 0.173, *p* = 0.012), indicating that stronger aftereffects are associated with better face recognition abilities. Although both perceptual biases are associated with face recognition ability, there was no significant relationship between serial dependence strength and face identity aftereffects (*r* = 0.041, *p* = 0.556) and while the relationship between serial dependence tuning and face identity aftereffects was small and positive (*r* = 0.100, *p* = 0.151), it was also not significant.

To determine whether serial dependence strength, serial dependence tuning, and face identity aftereffects were significant, unique predictors of face recognition abilities we used a multiple hierarchical regression (for regression statistics, see Table 3). The residualised car memory test scores were entered into the regression as the first step to estimate the amount of variance in face recognition ability that non-face recognition accounted for. This resulted in a statistically significant model, *F*(1, 207) = 19.077, *p* < 0.001. Serial dependence strength, serial dependence tuning, and face identity aftereffects were then entered into the regression in the second step, which also produced a statistically significant model, *F*(1, 204) = 10.091, *p* < 0.001. Examining the individual predictors revealed that non-face recognition, serial dependence tuning, and face identity aftereffects all made significant and unique contributions to face recognition ability. However, serial dependence strength was not found to be a significant, unique predictor of face recognition ability. Overall, this suggests that serial dependence tuning, and not strength, is a significant contributor to face recognition, independently of both non-face recognition ability and face identity aftereffects.

**Table 3:** Hierarchical Multiple Regression Analysis for Variables Predicting Face Recognition Ability from Object Recognition, Serial Dependence Strength, Serial Dependence Tuning, and Face Identity Aftereffects.

	*R*^2^	Δ*R*^2^	*β*	*p*	*sr*
Step 1	0.084	0.084			
CCMT_residuals			0.290	>0.001	0.290
Step 2	0.165	0.081			
CCMT_residuals			0.266	>0.001	0.263
SDFI_strength			−0.065	0.495	−0.044
SDFI_tuning			0.276	0.004	0.186
FIAE			0.142	0.029	0.141

Note. *N* = 209; SDFI = Serial Dependence of Facial Identity Task, FIAE = Face Ideneity Aftereffect Task, CCMT = Cambridge Car Memory Test.

## Discussion

Our results show that individual differences in serial dependence of facial identity are associated with variation in face recognition abilities. Both serial dependence strength and tuning showed significant associations, of small to moderate size^42^, with face recognition abilities. The relationship between serial dependence tuning and face recognition was further found to be significantly stronger than the relationship between this bias and object recognition. Serial dependence tuning was additionally found to be a significant and unique predictor of face recognition abilities, independently of both object recognition ability and face identity aftereffects. Overall, we found that the degree to which an individual showed stronger serial dependence for more similar over less similar faces predicted face recognition abilities.

The key aspect of these results is the finding that the selective use of serial dependence for highly similar faces is important to face recognition, and not simply the strength of the bias. This is important because previous research examining serial dependence has generally focused only on the strength of this bias and not the tuning, and no prior work has examined individual differences in the tuning of serial dependence. At the group level we found serial dependence of facial identity was tuned to the degree of similarity between successive stimuli, operating most strongly when stimuli are very similar. However, at the individual level there was substantial variation, with not all individuals showing this “narrow” tuning pattern. The association we found between the narrowness of tuning and face recognition ability suggests that serial dependence might only be functionally beneficial to face recognition when stimuli are similar. In contrast, integrating when stimuli are dissimilar might instead lead to greater error in recognition. Previous research has suggested that a broader tuning pattern, or assimilating despite larger differences between stimuli, results in greater error in estimation because the fine details needed for discrimination would be lost through integration^19^. This interpretation is consistent with our research, where broader tuning was associated with poorer face recognition abilities while narrower tuning was associated with better face recognition abilities.

The small to moderate effect sizes found in our study suggests that the association between serial dependence of facial identity tuning and face recognition abilities is comparable to that between face identity aftereffects and face recognition abilities, both in our current results and compared to previous research (c.f., Engfors, *et al*.^3^ *r* = 0.45 (corrected for attenuation) and Rhodes, *et al*.^4^ *r* = 0.17). Face identity aftereffects are argued to be important for face recognition abilities as they aid in the discrimination of individuals’ identities^4^. Here we find that in addition to discrimination, integration is also important for face recognition. This finding is similar to research suggesting that in face recognition, it is important to be able to both tell faces apart but also tell faces together, due to substantial within-person variability^43^. It may be that both serial dependence and adaptive coding are important for recognition, but which is optimal depends on the perceptual differences between the two faces and duration they are seen for. In contrast to serial dependence, which is strongest when two stimuli are more similar, face identity aftereffects instead increase in strength when similarity decreases^17^. Given we find that both of these perceptual biases are significant and unique predictors of face recognition abilities, in face recognition, it may therefore be best to integrate similar faces and discriminate dissimilar faces.

Our results showed no significant association between serial dependence of facial identity (strength or tuning) and face identity aftereffects. Both perceptual biases were also found to contribute independently to face recognition abilities. These results raise the possibility that serial dependence of facial identity and face identity aftereffects may be independent processes. However, we are unable to make strong conclusions regarding the independence of these perceptual biases because the reliability for both tasks is not ideal and there was a numerically small, although not significant, relationship between serial dependence tuning and face identity aftereffects.

Previously, research has suggested that the contribution of face identity aftereffects to face recognition can be considered relatively specific to face recognition and not general object recognition abilities^3–5^. We found similar results for serial dependence, in that serial dependence strength and tuning were significantly related to face recognition abilities but not general object recognition ability. For serial dependence tuning, this relationship was further found to be significantly stronger than the relationship between tuning and object recognition. Further, serial dependence tuning was still found to be a significant predictor of face recognition abilities when general object recognition was controlled for. If serial dependence of facial identity was a more general process that benefited all kinds of object recognition, we would have expected it to also be associated with object recognition. Given that we found no significant relationship between serial dependence and object recognition, our results suggest that serial dependence of facial identity, like face identity aftereffects, may be operating on the level of facial identity specifically, rather than operating on all stimuli in general.

Our results raise the possibility that serial dependence of facial identity may be altered in individuals with poorer face recognition abilities, such as those with prosopagnosia or autism, as has been found with face identity aftereffects^44,45^. It is unknown, however, whether the strength of serial dependence would be weakened in these individuals, or if instead they would show a different pattern of tuning of serial dependence, potentially using this bias more broadly. The findings from our study could therefore be expanded upon in future research by examining serial dependence in clinical populations with face processing difficulties.

Given our findings are based on more controlled stimuli, it is important for future research to further examine the relationship between serial dependence of facial identity and face recognition using more naturalistic images. Examining how this bias varies using multiple, naturalistic images of the same individual compared to different individuals will provide additional insight into the role of serial dependence of facial identity in face recognition, despite within-person variability.

It is also important to note that our estimate of serial dependence tuning was based on limited data points and so only a coarse estimate could be obtained. There are two implications. First, our results might be an underestimation of the relationship between tuning and face recognition abilities. Second, due to the relatively small number of increments assessed, linear, but not curvilinear functions were able to be fit to the current individual data. Previous research has used both linear regressions and curvilinear functions to examine serial dependence^27,33^. Linear regression provides an estimate of the effect of the previous stimulus on the current^19^ while curvilinear functions additionally capture the tendency for serial dependence to be stronger when consecutive stimuli are more similar than when they are less similar. Future studies aiming to precisely assess tuning would benefit from additional steps of difference between Face 1 and Face 2, to allow for curvilinear functions.

In conclusion, our study provides evidence that serial dependence of facial identity contributes to face recognition abilities. We find specifically that the extent to which an individual selectively uses serial dependence, the tuning of this bias, is important to face recognition. Our pattern of results suggests that strategic use of serial dependence, specifically for more similar faces, is beneficial for face recognition while using this bias for all faces, regardless of their similarity, is detrimental to face recognition abilities. Importantly, this relationship was specifically found for face recognition, suggesting that serial dependence of facial identity is operating at the level of facial identity rather than more general object processing levels. These findings raise important theoretical questions as to whether serial dependence of facial identity is altered in populations with weakened face recognition.

## Method

### Participants

Two hundred and forty-eight adults participated in the current study. Participants recruited were undergraduate students from the University of Western Australia who participated for course credit (*N* = 150) or adults recruited by invitation from student researchers who collected a subset of the data as a course requirement (*N* = 98). An a priori power analysis determined that a sample of 200 individuals would be sufficient to detect a ‘typical’ effect of *r* = 0.20 at 80% power^42^. Additional participants beyond the suggested sample size were tested due to the experiment being part of a course where student researchers collected data.

Participants were excluded from all analyses if they reported: being colour-blind (one of the tasks used coloured visual noise; *n* = 1); having a neurological disorder (*n* = 2); or not having lived in Australia or another Caucasian country for at least 10 years (*n* = 13) in an effort to ensure our sample had sufficient experience with our Caucasian face stimuli and minimise any effects of the own-race bias^46^. Additional participants were excluded due to poor testing conditions (e.g. excessive room noise, computer problems, excessive participant fatigue; *n* = 7) or because they were unable to learn the faces at the beginning of the serial dependence task (performing at less than 75% during training; *n* = 6). The final sample consisted of 219 participants (128 female, *M* = 20.05 years, *SD* = 2.79). For analyses examining face identity aftereffects, additional participants were excluded because they were unable to learn the faces at the beginning of the face identity aftereffect task (performing at less than 75% during training; *n* = 10), resulting in a final sample for these analyses of 209 participants (121 female, *M* = 20.07 years, *SD* = 2.84). All participants reported normal or corrected to normal vision and were aged between 17 and 33 years old.

### Tasks

#### Serial dependence of facial identity task

This task was based on Fischer and Whitney’s^18^ design which originally used Gabor patches.

#### Stimuli

**Figure 4:**
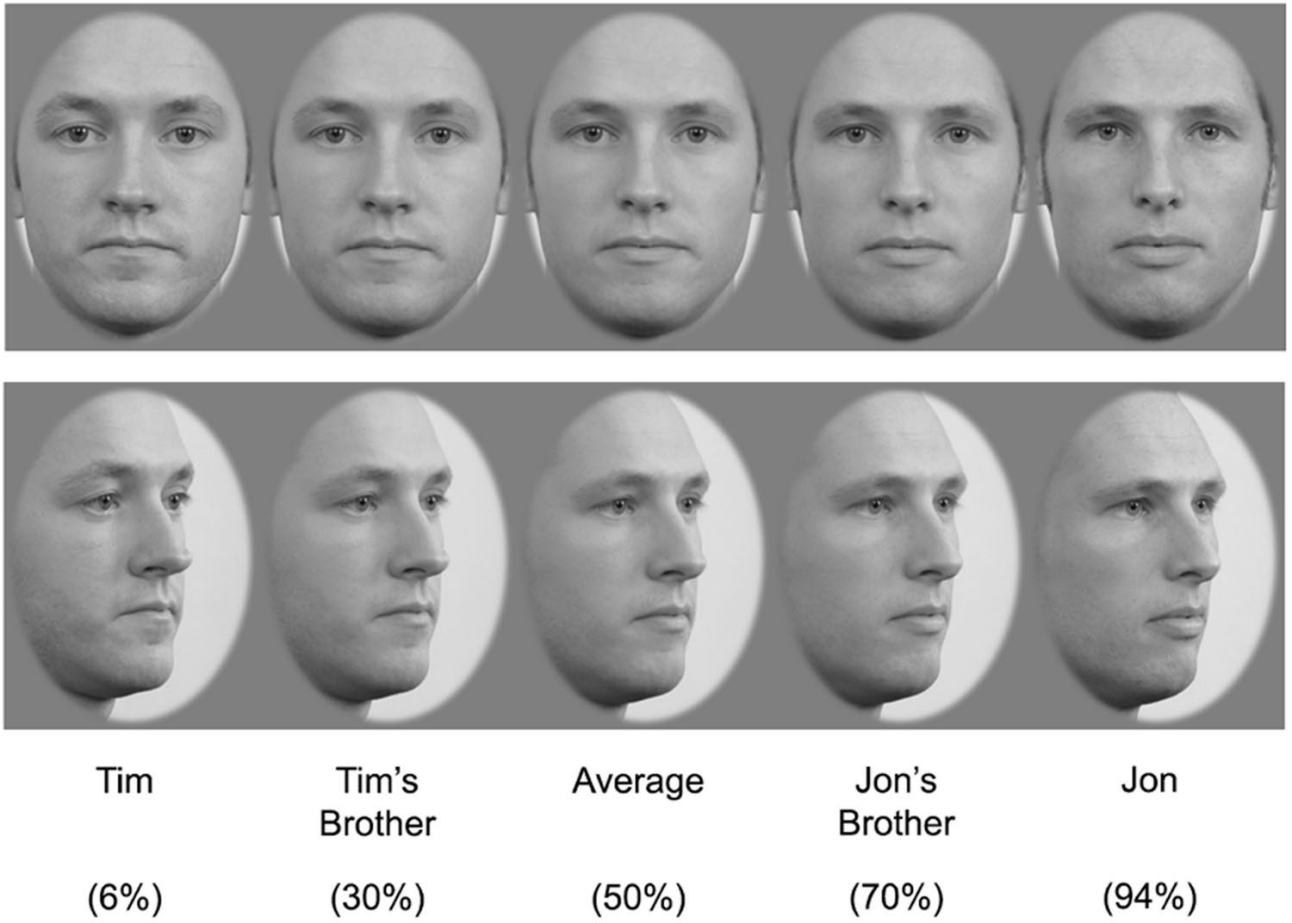
Male forward and left 1/4 profile facing identities learned by each participant and the average face (middle), which was a combination of 50% Jon and 50% Tim. The full-strength identities are at the extreme ends of the morph continuum while the siblings are closer to the average (±20% from the average). In this example, the percentages represent the percentage of the Jon identity in the morph (a higher percentage morph is a face that is more like Jon). Morph identities were created using images obtained with permission from the Radboud Faces Database^47^.

Pairs of identities selected by the authors to be easily discriminable, two male (‘Tim’ and ‘Jon’) and two female (‘Mel’ and ‘Sue’), were obtained from the Radboud Faces Database^47^. Each identity displayed a neutral expression, was masked with an oval aperture, and was shown in two different viewpoints (to reduce reliance on image cues to identify the faces), a left 1/4 profile and a frontal view, with consistent lighting in both viewpoints. Within each identity pair, and for each viewpoint, the two identities were morphed together, using Abrosoft FantaMorph 5 Deluxe (version 5.4.8), to produce a morph continuum of 23 faces, where each face morph differed from the next by 4%, between the endpoints of 6% to 94% (see Fig. 4).

#### Procedure

**Figure 5:**
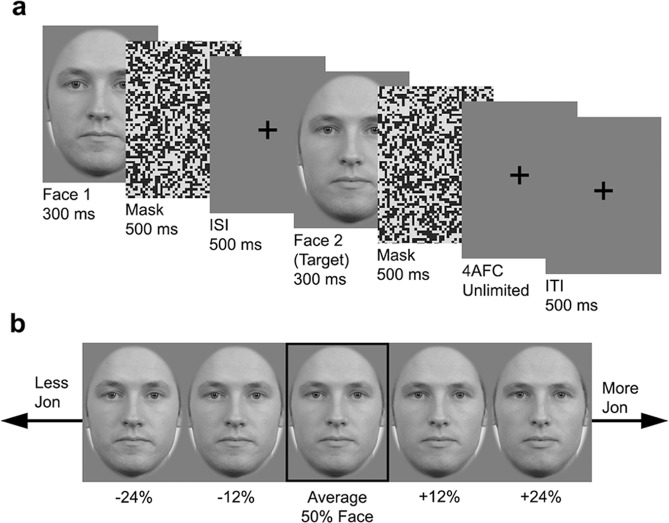
Experiment trial sequence and possible differences between Face 1 and Face 2. Figure (**a**) is a schematic showing one trial. On each trial, participants were presented with a face (300 ms), followed by a visual noise mask (500 ms) and inter-stimulus interval (500 ms). Participants are then presented with a second face (300 ms) that, following a visual noise mask (500 ms), they are asked to identify. Following their response, there was an inter-trial interval (500 ms). In Fig. (**b**) the centre face in the black box is the average 50% face which could appear as Face 2. The images surrounding it are the four possible Face 1 stimuli for when Face 2 was the average 50% face. The two faces to the left are less Jon (−24% or −12%) and the two faces to the right are more Jon (+12% and +24%). In this example, it would be expected that the proportion of Jon responses to the average 50% centre face would decrease when Face 1 is less Jon and increase when Face 1 is more Jon. Morph identities were created using images obtained with permission from the Radboud Faces Database^47^.

To tap into identity processing, participants needed to be familiar with the identities at both viewpoints before beginning the serial dependence task. Participants were initially trained to identify the four target faces as well as their ‘siblings’ who were weaker strength versions of these targets (details in Supplementary Methods). In each trial of the serial dependence task, a face was presented for 300 ms, followed by a 500 ms noise mask, a 500 ms interstimulus interval, and then a second (target) face for 300 ms, followed by a 500 ms noise patch (see Fig. 5a). Participants were asked to identify the second face by pressing one of the four labelled keys. A fixation cross was presented for 500 ms prior to the next trial beginning. Gender and viewpoint did not vary within a trial. Half of the trials used male faces and within each gender, half of the trials were forward facing. There were 176 trials, presented in a pseudorandom order so that the identity of the target faces was not the same for more than four consecutive trials. Each participant completed the same order to remove any potential influence of trial order on an individual’s performance. The trials were divided into 4 blocks of 44 trials to provide participants with regular breaks.

Within each trial, Face 2 was one of the 11 faces from the centre of each morph continuum that ranged between 30% to 70%. On each trial, Face 1 differed from Face 2 in one of four ways: −24%, −12%, +12%, or +24% (see Fig. 5b for example), with each of these differences occurring in a quarter of the trials. Face 1 could be any of the 23 morphs between the two identities.

The first face subtended an average visual angle of 9.1° (v) × 6.7° (h) when viewed from a distance of approximately 60 cm. The target faces were smaller, 8.2° (v) × 6.0° (h), than the first face to reduce low-level retinotopic effects. The visual noise masks were 16 unique, binary noise masks that were luminance balanced relative to the grey screen and scaled to match the minimum and maximum luminance of the faces used within the study. Each visual noise mask measured 12° (v) x 12° (h) and were included to reduce the visible persistence of the test stimuli.

##### Reliability

The reliability for the strength of serial dependence was calculated by correlating the strength of serial dependence for each gender at each viewpoint, calculating the average correlation, and then applying the Spearman-Brown correction to account for the reduced trial numbers relative to the task overall. Reliability for the estimation of serial dependence strength (*ρ’* = 0.47) is consistent with previous research examining perceptual biases^3–5^. The serial dependence tuning value was calculated using difference scores, therefore to calculate the reliability of tuning, a formula was applied to account for the correlation (*r* = 0.49) between the two conditions (similar and dissimilar faces) being compared^48^. Reliability for the tuning of serial dependence score (*ρ*_*dd’*_ = 0.13) was poor, partially due to the large correlation between the serial dependence for similar and dissimilar faces^48^.

#### **Face identity aftereffect task**

This task has been widely used to measure face identity aftereffects^5^. We used Rhodes *et al*.’s^5^ version of this task. Participants were trained to identify four male targets (100% identity strength) and their ‘brothers’ (weaker versions, 30% identity strength). In the aftereffect task, participants viewed an adapting anti-face (8000 ms; four 2000ms exposures separated by 150 ms blank interstimulus intervals), followed by an interstimulus interval (150 ms), and finally a test face (400 ms) that they had to identify. The task consisted of 48 trials. In 32 trials, the antiface was followed by a weak test face (15%). In half of these trials, the antiface that participants adapted to was a match to the test face (e.g. adapt antiTed, test 15% Ted). The other half were mismatch trials (e.g. adapt antiTed, test 15% Dan). Adaptation to an antiface facilitates identification of a test face in match, but not mismatch, trials. The face identity aftereffect was calculated as accuracy on match trials minus accuracy on mismatch trials. To maintain motivation, in the remaining 16 trials the test faces were a high identity strength (90%). These trials were not used to calculate the aftereffect. To help ensure attention to the anti-face, on half of the trials an asterisk appeared in one of the interstimulus intervals. Participants were asked to report whether the asterisk was present or not for each trial. To calculate the reliability of the face identity aftereffect task, a formula was applied to account for the correlation (*r = *0.03) between the two conditions that produced the difference score (match and mismatch trials). Reliability for the face identity aftereffect task (*ρ*_*dd’*_ = 0.43) was consistent with previous research examining face identity aftereffects^3–5^.

#### **Cambridge face memory test (CFMT)**

The CFMT^37^ is a widely used measure of face recognition ability. In this task, participants learn six male faces from three different viewpoints. Recognition of these faces is then tested using a 3AFC format (one target and two distractors) over three stages. In the first stage (18 trials), test faces were identical to the images initially learned. In the second stage (30 trials), test faces varied in viewpoint and lighting. The third stage (24 trials) was identical to the second, except with visual noise added. Percentage accuracy on the CFMT was used as an estimate of face recognition ability. Reliability for the CFMT was calculated using Cronbach’s alpha reliability between the three subscales, (see Engfors, *et al*.^3^). Reliability has been well established for the CFMT^38^ and our results (*α* = 0.66) are consistent with previous research^3^.

#### **Cambridge car memory test (CCMT)**

The CCMT^39^ is a measure of non-face object recognition ability. The procedure and format of this task is identical to that of the CFMT, however cars are used instead of faces as stimuli. Percentage accuracy on the CCMT was used as an estimate of non-face recognition ability. Reliability for the CCMT is also well established^39^ and was calculated as above. Reliability (*α* = 0.77) was consistent with previous research^3^.

### Procedure

Participants were tested individually in a 1–1.5-hour session with regular breaks. After written and informed consent had been obtained, participants were seated in a quiet cubicle approximately 60 cm from an iMac. Participants first completed the serial dependence of facial identity task (programmed and presented via SuperLab 4.5, Cedrus Corp) followed by the CFMT (original java version). This was followed by a 15-minute break in which participants completed demographic information and questionnaires (Autism Spectrum Quotient and Glasgow Sensory Questionnaire; results will be reported elsewhere). Participants then completed the face identity aftereffect task (SuperLab 4.5) and the CCMT (original java version) and finally were debriefed at the end of the session. The experimental procedure was approved by the University of Western Australia’s Human Research Ethics Committee and the experiment was performed in accordance to their guidelines and regulations.

## Supplementary information

Supplementary Methods

## Data Availability

The data sets created and analysed during the current research are available from the corresponding author on reasonable request.
