# Supplementary material for: Individual Differences in Serial Dependence of Facial Identity are Associated with Face Recognition Abilities
Source: Sci Rep. 2019 Dec 2;9:18020. doi: 10.1038/s41598-019-53282-3 (PMC6888837; doi:10.1038/s41598-019-53282-3)
Supplement: Supplementary file 1 — Supplementary Methods [file 41598_2019_53282_MOESM1_ESM.docx]

# Individual Differences in Serial Dependence of Facial Identity are Associated with Face Recognition Abilities

Kaitlyn Turbett*, Romina Palermo, Jason Bell, Jessamy Burton, and Linda Jeffery

School of Psychological Science, University of Western Australia, Crawley, WA, Australia

* Corresponding author: kaitlyn.turbett@research.uwa.edu.au; School of Psychological Science, M304, University of Western Australia, 35 Stirling Highway Crawley, WA 6009, Australia

**Supplementary Methods**

**Serial dependence of facial identity task – training phase**.

To tap into identity processing, participants needed to be familiar with the identities at both viewpoints before beginning the serial dependence task. In the training phase, participants were first presented with the four identities (first the male pair and then the female pair) at full-strength (94%) for an unlimited amount of time. They were instructed to press the spacebar to move onto the next stage when they felt ready. Following this, participants were required to identify individually presented faces by pressing one of four labelled keys that corresponded to the four identities. Faces were initially presented for an unlimited amount of time, and then for 300ms once participants progressed. In order to progress to each stage, participants needed to correctly identify 75% of the faces presented to them, based on a block of 8 faces. If a participant was unable to correctly identify at least 75% of the faces presented, the training stage repeated again (maximum of 3 repetitions). After having learnt the full-strength faces, participants were presented with their ‘siblings’ (faces ±20% from the average face) for an unlimited amount of time (for an example, see Figure 3). These faces were introduced to the participants as ‘siblings’ to highlight the similarity between each of the four original identities and their corresponding weaker strength morph, as has been done in previous face identity aftereffect research^1^. This stage progressed in the same way as the full-strength learning phase, with participants presented with either a full-strength or sibling face individually, first for an unlimited amount of time and then for 300ms once participants performed at or above 75%. Performance was calculated based on blocks of 16 faces. On average, participants performed at 93.7% accuracy in the training phase of this task. Of the 219 participants 75.4% did not need to repeat a training stage, 14.6% required one repetition of a training stage, 7.3% required two repetitions, and 2.7% required between 3 to 5 repetitions.

**References**

1 Jeffery, L. *et al.* Distinguishing norm-based from exemplar-based coding of identity in children: evidence from face identity aftereffects. *J. Exp. Psychol. Hum. Percept. Perform.* **37**, 1824-1840, doi:10.1037/a0025643 (2011).
